# High-resolution GPS tracking reveals sex differences in migratory behaviour and stopover habitat use in the Lesser Black-backed Gull *Larus fuscus*

**DOI:** 10.1038/s41598-018-23605-x

**Published:** 2018-03-29

**Authors:** Jan M. Baert, Eric W. M. Stienen, Brigitte C. Heylen, Marwa M. Kavelaars, Roland-Jan Buijs, Judy Shamoun-Baranes, Luc Lens, Wendt Müller

**Affiliations:** 1 Behavioral Ecology and Ecophysiology Research group, University of Antwerp, Universiteitsplein1, 2610 Antwerp, Belgium; 2 Terrestrial Ecology Unit (TEREC), Ghent University, K.L. Ledeganckstraat 35, 9000 Ghent, Belgium; 3 Research Institute for Nature and Forest (INBO), Havenlaan 88 box 73, 1000 Brussels, Belgium; 4 Buijs Eco Consult B.V., Philips van Dorpstraat 49, 4698 RV Oud-Vossemeer, The Netherlands; 5 Institute for Biodiversity and Ecosystem Dynamics (IBED), University of Amsterdam, P.O. Box 94248, 1090GE Amsterdam, The Netherlands

## Abstract

Sex-, size- or age-dependent variation in migration strategies in birds is generally expected to reflect differences in competitive abilities. Theoretical and empirical studies thereby focus on differences in wintering areas, by which individuals may benefit from avoiding food competition during winter or ensuring an early return and access to prime nesting sites in spring. Here, we use GPS tracking to assess sex- and size-related variation in the spatial behaviour of adult Lesser Black-backed Gulls (*Larus fuscus*) throughout their annual cycle. We did not find sex- or size-dependent differences in wintering area or the timing of spring migration. Instead, sexual differences occurred prior to, and during, autumn migration, when females strongly focussed on agricultural areas. Females exhibited a more protracted autumn migration strategy, hence spent more time on stopover sites and arrived 15 days later at their wintering areas, than males. This shift in habitat use and protracted autumn migration coincided with the timing of moult, which overlaps with chick rearing and migration. Our results suggest that this overlap between energy-demanding activities may lead females to perform a more prolonged autumn migration, which results in spatiotemporal differences in foraging habitat use between the sexes.

## Introduction

Long-distance migration has evolved in many genera of birds to cope with spatio-temporal fluctuations in the environment^1,2^. This enables migratory species to complete their annual cycle under favourable conditions, for example by taking advantage of seasonal peaks in food availability^3^. Migration is, however, an energetically costly endeavour^4–6^ known to trade-off against other energy-demanding activities in a bird’s annual cycle, in particular breeding and moulting^7–9^. These trade-offs in energy allocation have been shown to vary among individuals within a population in relation to experience^10,11^, sexual maturity^12^, size^13^ or social dominance^14^. Such age-, sex-, or size-dependent variation in the timing of migration, choice of wintering areas, or migration routes, is commonly referred to as ‘differential migration’^15^. Several hypotheses have been proposed to explain how differential migration might arise^15^. Under the body size hypothesis^15^, it is predicted that larger birds winter closer to the breeding areas as they are better able to sustain colder winter temperatures. Alternatively, differential migration may arise because of a sorting in body size across a habitat quality gradient, as smaller individuals be pushed out of favourable habitats by more competitive, larger birds (social dominance hypothesis)^16^. Moreover, if one sex experiences stronger competition for breeding resources, it would benefit from arriving at the breeding sites earlier, and as consequence, from wintering closer to the breeding area, starting spring migration earlier and/or migrating faster (arrival time hypothesis)^17^.

Differential migration can only persist if the benefits of alternative migration strategies offset the associated costs. Migration is generally considered as a costly stage within the annual cycle^18^, as it requires time and energy or increases the risk of predation. These costs should be outweighed by the benefits of wintering in favourable areas or of a timely arrival at wintering or breeding sites. Hence, the dominant view has long been that observed variation in migration strategies results from trade-offs between time, energy and safety that minimise the cost of migration^5,19,20^. However, an increasing number of tracking studies revealed that migration routes can include significant detours and may vary substantially according to age, sex and weather conditions^12,13,21^. Migration studies have, therefore, shifted towards a life-history perspective in an attempt to understand the trade-offs and carry-over effects within the annual cycle that may underlie the observed variation in migration strategies^18,22,23^, and how these trade-offs and effects may vary with internal or external factors such as age, sex and fluctuations in habitat quality or weather conditions^12,21,24–27^. Understanding variation in life-history strategies in migratory species thus hinges on the ability to track individuals throughout their annual cycle. Large northern hemisphere gulls (*Larus* spp.) are an interesting model to study differential migration. Weighing over 500 g, these birds can readily be fitted with GPS trackers without any significant impact on their performance or survival^28^. Moreover, individuals from a single mixed breeding colony often exhibit both strong inter- and intraspecific variation in migration strategies^29–31^.

In this study, we focussed on populations of Lesser Black-backed Gulls (mixture of *Larus fuscus graellsii and Larus fuscus intermedius*) breeding along the south-eastern part of the North Sea. Previous ring recoveries have exposed a large degree of intraspecific variability in wintering sites within and among these populations, with some birds wintering as close as Northern France or Southern England, while others migrate as far south as Senegal^32^. Moreover, GPS tracking studies have revealed that birds do not fly directly to their wintering area, but make significant detours in autumn with frequent stopovers^30^. Lesser Black-backed Gulls exhibit a limited size dimorphism with males being, on average, larger than females, albeit with considerable overlap between sexes^29^. Hence, differential migration based on size, as predicted by the body size and social dominance hypotheses^15,16^, may result in sex-dependent migration strategies. However, previous studies have demonstrated that neither sex nor size significantly explain the high variability in migration routes or wintering areas in this species^30–32^. In addition, as males play a dominant role in establishing and defending nesting sites, they can be expected to benefit most from an earlier arrival in spring^33^. However, ringing recoveries revealed that differences in the timing of migration only occur during autumn migration, with males generally arriving on their wintering site before females, despite their simultaneous departure from the breeding area^32^.

Here, we used GPS tracking to study intraspecific variability in spatial behaviour at high spatio-temporal resolution throughout the annual cycle. Since Lesser Black-backed Gulls are known to make significant detours from the shortest migration routes, particularly during autumn migration when they spend considerable time at stopover sites^30,31^, we made a distinction between summer, winter, migration (i.e. directional flight to/from the wintering sites) and stopover (i.e. foraging during migration) bouts. The tracking data of 22 male and 26 female Lesser Black-backed Gulls breeding in the Southern Bight of the North Sea, that jointly completed 150 migration routes between 2013 and 2017, were analysed. We investigated to what extent differences in distance between wintering and breeding areas, in timing of migration, and in habitat use throughout the year, varied according to size and sex.

## Results

### Migration strategies

**Figure 1:**
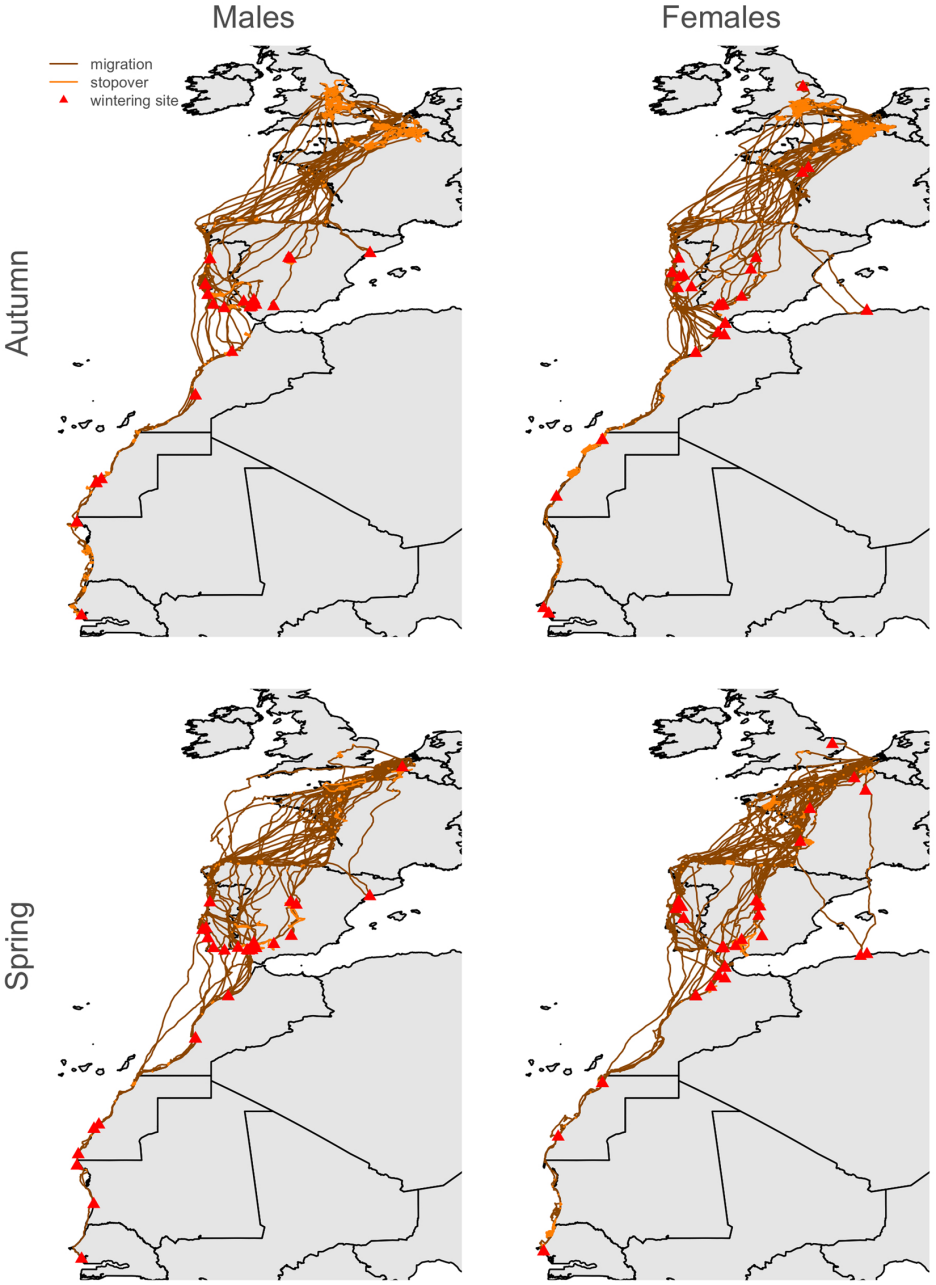
Autumn (n = 70) and spring (n = 80) migration routes of 48 Lesser Black-backed Gulls (22 males, 26 females). A distinction is made between migration trajectories (i.e. directional flight to wintering area, brown lines) and stopover trajectories (i.e. foraging during a stopover, orange lines). Wintering areas are indicated as red triangles. Maps were generated using the Maps package in R^61,64^.

**Figure 2:**
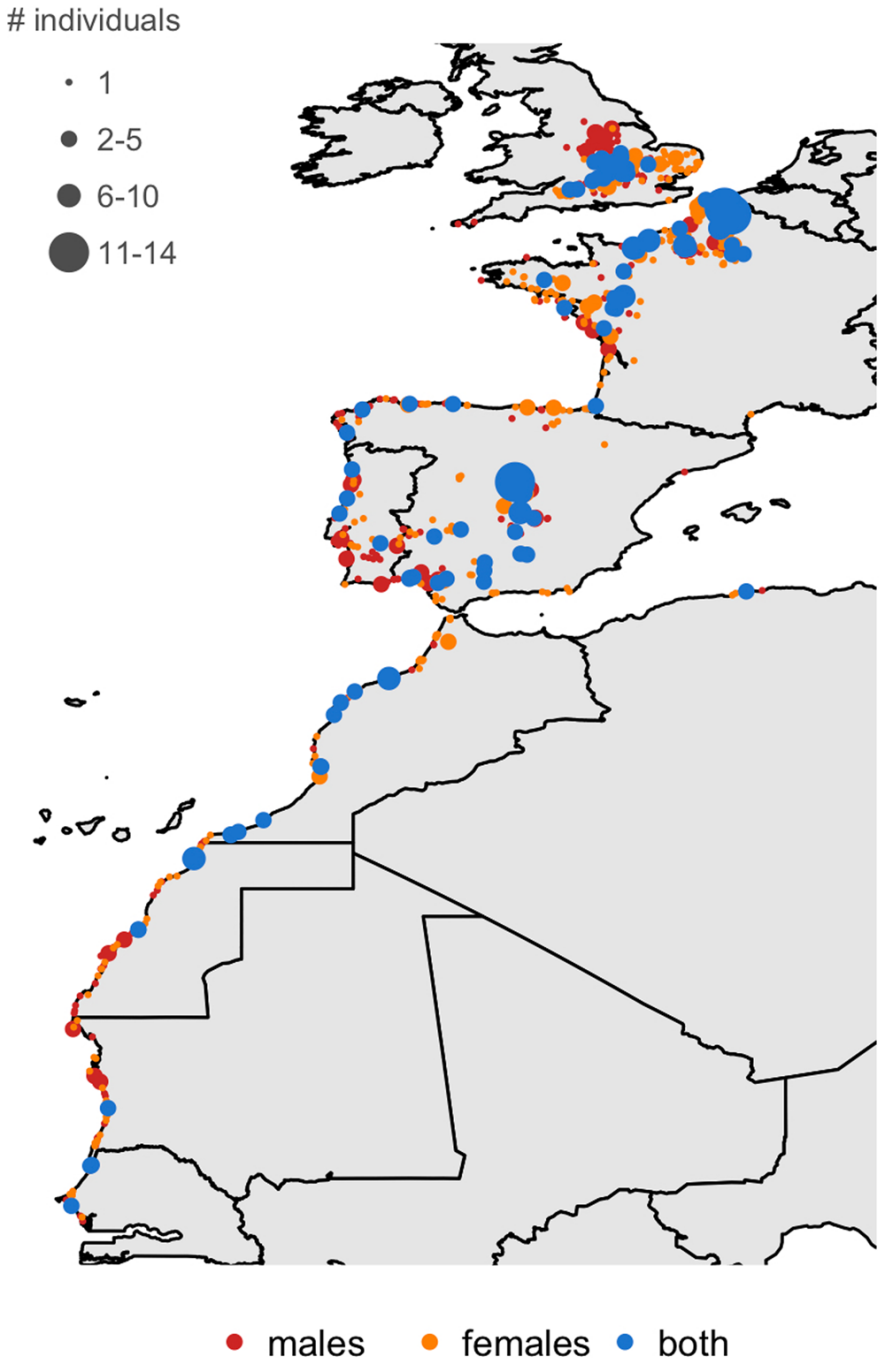
Migration stopover sites for 48 Lesser Black-backed Gulls. Sites used by males are indicated in red, sites used by females are indicated in light orange and sites used by both sexes are indicated in blue. The size of the dot is proportional to the number of individuals visiting a particular site. Maps were generated using the Maps package in R^61,64^.

**Figure 3:**
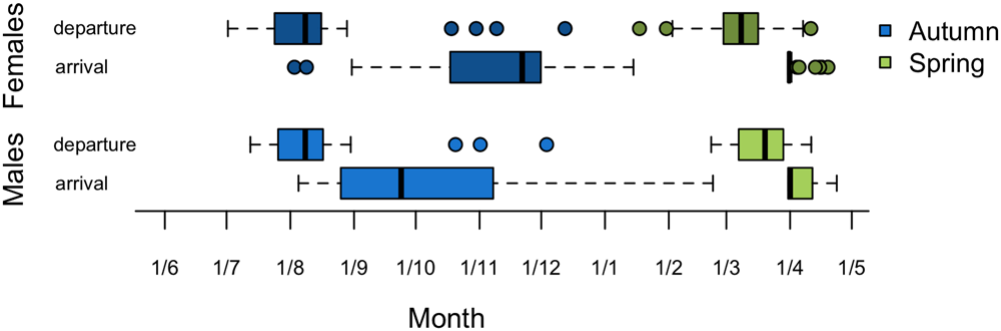
Timing of autumn and spring migration in male and female Lesser Black-backed Gulls. Departure and arrival dates are given as boxplots. Boxes and whiskers correspond to quartiles and maximal 1.5 the interquartile range. Note that migration periods are indicated with darker colours in females and lighter colours in males, in correspondence with Fig. 4.

The wintering areas of tracked Lesser Black-backed Gulls extended from the UK to Senegal (Fig. 1). Average distances between breeding and wintering areas did not vary significantly according to sex or body size (Table 1). Males and females used similar migration routes (Fig. 1) and stopover sites (Fig. 2), with a high inter-individual variation. Although both sexes commenced autumn migration at the same time, males tended to arrive at their wintering grounds 15 days earlier on average than females (Fig. 3, Table 1). During autumn migration, females therefore spent more time on average at stopover sites, in particular in the UK and northern France (Fig. 1). The timing of autumn migration was independent of prior breeding success (Fig. S15). Males that wintered further from the breeding area, however, arrived later at their wintering sites than males wintering more nearby (Table 1). No significant sex differences were observed in the onset or end of spring migration (Table 1). Instead, the onset and end of spring migration significantly varied in relation to body size. Larger birds departed earlier and arrived later than smaller ones (negative and positive slope of the body size x migration distance interaction respectively, Table 1), while birds wintering further away arrived earlier at the breeding sites (Table 1).

**Table 1:** Gaussian mixed effects models of (i) migration distance in relation to sex and body size, and (ii) the onset and end of spring and autumn migration in relation to sex, body size and migration distance. All models included year as random effect. Non-significant interactions were removed from each model.

	F-statistic	d.f.	p-value	Estimated marginal mean/coefficient ± s.e.
**Migration distance (km)**				
Sex	0.14	1,143	0.69	
Male				1941 ± 257
Female				2071 ± 230
Body size	2.1147	1,143	0.15	20.33 ± 13.98
**Onset of autumn migration (Julian day)**				
Sex	0.77	1,63	0.38	
Male				219 ± 9
Female				234 ± 11
Body size	0.76	1,63	0.39	−0.65 ± 0.73
Migration distance	3.96	1,63	0.05	−0.0081 ± 0.0041
**End of autumn migration (Julian day)**				
Sex	4.22	1,62	0.04	
Male				289 ± 14
Female				304 ± 12
Body size	1.14	1,62	0.29	−1.01 ± 0.94
Migration distance	0.28	1,62	0.59	
Sex x migration distance	6.28	1,62	0.02	
Male				−0.0035 ± 0.0068^*^
Female				0.0264 ± 0.011
**Onset of spring migration (Julian day)**				
Sex	0.19	1,71	0.66	
Male				92 ± 4
Female				54 ± 16
Body size	0.05	1,71	0.82	0.14 ± 0.64
Migration distance	5.01	1,71	0.03	
Body size x migration distance	5.15	1,71	0.03	−0.00075 ± 0.00033
Sex x migration distance	8.83	1,71	0.01	
Male				0.16 ± 0.073
Female				0.18 ± 0.078
**End of spring migration (Julian day)**				
Sex	0.60	1,72	0.44	
Male				91 ± 2
Female				93 ± 2
Body size	5.21	1,72	0.03	−0.35 ± 0.15
Migration distance	8.44	1,72	0.004	−0.041 ± 0.014
Body size x migration distance	10.07	1,72	0.002	0.002 ± 0.0006

^*^p < 0.05.

### Habitat use

**Figure 4:**
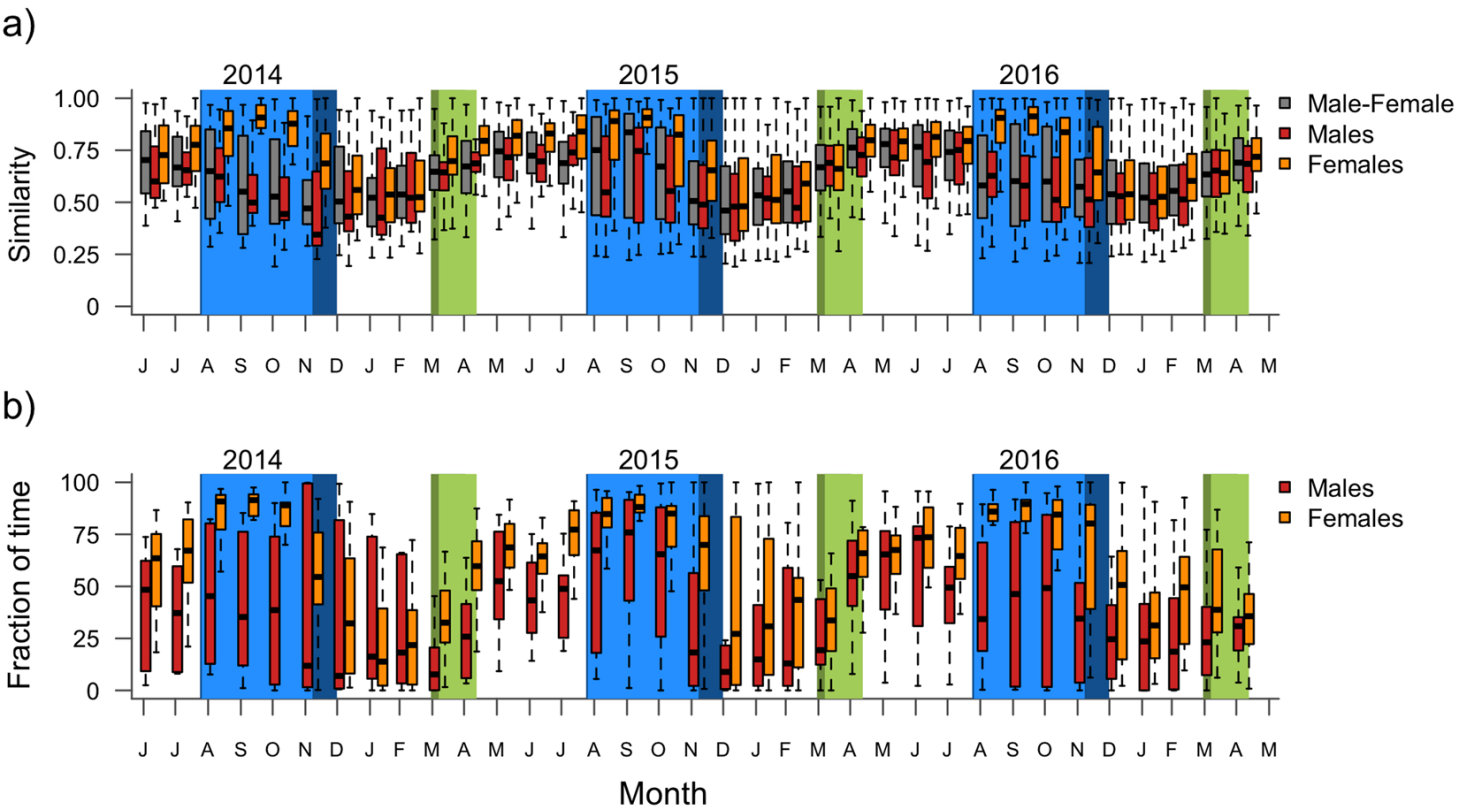
(**a**) Inter- and intra-sex similarity in habitat use. (**b**) Fraction of time spent in agricultural habitats. Migration periods of males are indicated in lighter colours than those of females. Note that migration in both sexes completely overlaps (Fig. 3). Boxes and whiskers correspond to quartiles and maximal 1.5 the interquartile range.

Lesser Black-backed Gulls exhibited distinctive annual cycles in habitat use, which were more similar within than between sexes (Table 2, Fig. 4). These temporal dynamics, however, only differed between sexes with regards to agricultural and marine habitats, as evident from significant sex x month interactions (Table 2). Seasonal patterns in presence in urban and freshwater habitats did not differ between sexes (Table 2). Sex differences in habitat use were most pronounced at the end of the breeding season and during autumn migration (Fig. 4). The analysis of individual variation in habitat use revealed that between June and November, females were more uniform in their habitat use compared to the rest of the year, whereas individual differences between males remained consistent throughout the year (Table 3). Reduced variability in habitat use in females during this period appeared to be driven by a stronger dependence on agricultural habitats (Fig. 4b and Table 4). While males tended to show a similar dependency on agricultural habitats between June and October (Table 4), variation in habitat use among males remained high throughout the year (Table 3, Fig. 4a). In both sexes, increased use of agricultural habitats was generally offset by a decreased use of marine habitats.

**Table 2:** Gaussian mixed effects models of (i) similarity in habitat use, and (ii) use of agricultural, urban, freshwater and marine habitats. The model for similarity in habitat use included month and pair type (Male-Female, Male-Male, Female-Female) of individuals being compared as fixed effects, and year and the pair identity as random effects. Models of habitat use included sex and month as fixed effects, as well as individual and year as random effects. Non-significant effects were removed from each model. Estimated marginal means for these models are reported in Table 3 (similarity in habitat use) and 4 (other models).

	F-statistic	d.f.	p-value
**Similarity in habitat use**			
Pair type	1.277	2,1051	0.28
Month	87.86	11,13226	<0.001
Pair type × month	18.88	22,13226	<0.001
**Use of agricultural habitats**			
Sex	1.04	1,46	0.31
Month	15.08	11,930	<0.001
Sex × month	2.68	11,930	0.002
**Use of urban habitats**			
Sex	18.21	1,46	<0.001
Month	11.85	11,930	<0.001
**Use of freshwater habitats**			
Sex	1.45	1,46	0.234
Month	14.54	11,930	<0.001
**Use of marine habitats**			
Sex	0.66	1,46	0.42
Month	11.68	11,930	<0.001
Sex × month	2.44	11,930	0.005

**Table 3:** Estimated mean monthly similarity (±s.e.) in habitat use within and between sexes, obtained from a Gaussian mixed effects model that included month and pair type (Male-Female, Male-Male, Female-Female) as fixed effects, and year and pair identity as random effects (see Table 2). Similarity was calculated as the Bray-Curtis similarity index, with higher values representing higher similarity in habitat use between individuals. Significance of estimated differences between pair types was assessed from Tukey’s HSD tests (5% significance level) for each month. Identical letters and numbers indicate non-significant differences between pair type and months, respectively. Significance of differences between months was tested against the January value.

	Male-Female	Male-Male	Female-Female
January	0.542 ± 0.007 ^A,1^	0.531 ± 0.012 ^A,1^	0.555 ± 0.009 ^A,1^
February	0.569 ± 0.007 ^A,2^	0.551 ± 0.012 ^A,1^	0.584 ± 0.009 ^A,1^
March	0.647 ± 0.007 ^A,2^	0.638 ± 0.012 ^A,1^	0.661 ± 0.009 ^A,1^
April	0.710 ± 0.007 ^A,2^	0.677 ± 0.012 ^A,1^	0.760 ± 0.009 ^B,2^
May	0.736 ± 0.007 ^A,2^	0.712 ± 0.011 ^A,1^	0.768 ± 0.009 ^B,1^
June	0.722 ± 0.006 ^A,2^	0.683 ± 0.010 ^B,1^	0.780 ± 0.008 ^C,2^
July	0.709 ± 0.007 ^A,2^	0.727 ± 0.010 ^A,1^	0.817 ± 0.008 ^B,2^
August	0.648 ± 0.007 ^A,2^	0.628 ± 0.011 ^A,1^	0.828 ± 0.009 ^B,2^
September	0.654 ± 0.007 ^A,2^	0.609 ± 0.012 ^A,1^	0.833 ± 0.009 ^B,2^
October	0.625 ± 0.007 ^A,2^	0.579 ± 0.012 ^A,1^	0.744 ± 0.009 ^B,2^
November	0.550 ± 0.007 ^A,1^	0.539 ± 0.012 ^A,1^	0.655 ± 0.009 ^B,1^
December	0.543 ± 0.008 ^A,1^	0.524 ± 0.013 ^A,1^	0.560 ± 0.010 ^A,1^

**Table 4:** Estimated mean fraction (±s.e.) of time spent in agricultural, urban, freshwater or marine habitats per month, derived from linear mixed effect models including sex and month as fixed effects, and individual and year as random effects. Significance of differences between sexes was assessed from Tukey’s HSD tests (5% significance level) for each month. Identical letters and numbers indicate non-significant differences between sexes and months, respectively. Significance between months was tested against the January value.

	Agricultural habitats		Urban habitats		Freshwater habitats		Marine habitats	
	Males	Females	Males	Females	Males	Females	Males	Females
January	0.286 ± 0.050 ^A,1^	0.355 ± 0.044 ^A,1^	0.243 ± 0.030 ^A,1^	0.113 ± 0.026 ^B,1^	0.132 ± 0.019 ^A,1^	0.144 ± 0.017 ^A,1^	0.338 ± 0.044 ^A,1^	0.385 ± 0.038 ^A,1^
February	0.267 ± 0.050 ^A,1^	0.385 ± 0.044 ^A,1^	0.268 ± 0.029 ^A,1^	0.169 ± 0.026 ^B,1^	0.161 ± 0.019 ^A,1^	0.122 ± 0.017 ^A,1^	0.305 ± 0.044 ^A,1^	0.321 ± 0.038 ^A,2^
March	0.234 ± 0.049 ^A,1^	0.355 ± 0.044 ^B,2^	0.257 ± 0.029 ^A,1^	0.162 ± 0.025 ^B,1^	0.096 ± 0.019 ^A,1^	0.083 ± 0.016 ^A,1^	0.414 ± 0.043 ^A,1^	0.397 ± 0.038 ^A,1^
April	0.390 ± 0.048 ^A,1^	0.522 ± 0.043 ^B,2^	0.335 ± 0.028 ^A,2^	0.262 ± 0.025 ^B,2^	0.029 ± 0.019 ^A,2^	0.016 ± 0.017 ^A,2^	0.247 ± 0.043 ^A,1^	0.197 ± 0.037 ^A,2^
May	0.509 ± 0.049 ^A,1^	0.590 ± 0.044 ^A,2^	0.321 ± 0.029 ^A,2^	0.238 ± 0.026 ^B,2^	0.022 ± 0.019 ^A,2^	0.008 ± 0.015 ^A,2^	0.142 ± 0.043 ^A,2^	0.160 ± 0.039 ^A,2^
June	0.489 ± 0.048 ^A,1^	0.643 ± 0.041 ^B,2^	0.259 ± 0.028 ^A,1^	0.165 ± 0.024 ^B,1^	0.025 ± 0.018 ^A,2^	0.008 ± 0.016 ^A,2^	0.233 ± 0.041 ^A,1^	0.139 ± 0.038 ^A,2^
July	0.423 ± 0.048 ^A,2^	0.656 ± 0.041 ^B,2^	0.353 ± 0.028 ^A,2^	0.195 ± 0.024 ^B,2^	0.025 ± 0.018 ^A,2^	0.007 ± 0.016 ^A,2^	0.200 ± 0.043 ^A,1^	0.064 ± 0.036 ^A,2^
August	0.431 ± 0.048 ^A,2^	0.801 ± 0.042 ^B,2^	0.224 ± 0.029 ^A,1^	0.114 ± 0.025 ^B,1^	0.053 ± 0.019 ^A,2^	0.011 ± 0.016 ^A,2^	0.257 ± 0.043 ^A,2^	0.065 ± 0.037 ^B,2^
September	0.478 ± 0.049 ^A,2^	0.825 ± 0.043 ^B,2^	0.215 ± 0.029 ^A,1^	0.107 ± 0.025 ^B,1^	0.081 ± 0.019 ^A,2^	0.020 ± 0.016 ^A,2^	0.223 ± 0.044 ^A,2^	0.044 ± 0.039 ^B,2^
October	0.458 ± 0.050 ^A,2^	0.741 ± 0.044 ^B,2^	0.172 ± 0.030 ^A,1^	0.077 ± 0.025 ^B,1^	0.075 ± 0.019 ^A,2^	0.074 ± 0.016 ^A,2^	0.281 ± 0.044 ^A,2^	0.106 ± 0.038 ^B,2^
November	0.332 ± 0.050 ^A,2^	0.606 ± 0.044 ^B,2^	0.182 ± 0.030 ^A,1^	0.061 ± 0.026 ^B,1^	0.127 ± 0.019 ^A,1^	0.101 ± 0.017 ^A,1^	0.358 ± 0.044 ^A,1^	0.230 ± 0.039 ^B,2^
December	0.279 ± 0.051 ^A,1^	0.431 ± 0.045 ^A,2^	0.199 ± 0.030 ^A,1^	0.085 ± 0.026 ^B,1^	0.140 ± 0.019 ^A,1^	0.133 ± 0.017 ^A,1^	0.382 ± 0.045 ^A,1^	0.350 ± 0.039 ^A,1^

## Discussion

High-resolution GPS tracking revealed sexual differences in spatial behaviour in our study population of Lesser Black-backed Gulls during the late breeding season and autumn migration, but not during winter. Although males and females departed simultaneously and used similar migration routes (Figs 1 and 2), it took females 10 days longer on average to reach their wintering areas (Fig. 3). Moreover, we found that these differences in the timing of autumn migration coincided with shifts in habitat use. From the late breeding season until the end of autumn migration, females almost exclusively resided in agricultural habitats, whereas males frequented a wider variety of habitats during autumn (Fig. 4). By lingering longer at stopover sites and specialising on agricultural food sources during stopovers, females were both spatially and temporally segregated from males.

Differential migration is generally hypothesized to either reduce resource competition in winter or increase breeding success in spring due to access to prime nesting sites^15,34,35^. However, in our study species, ringing and tracking studies have so far provided little empirical support for the underlying drivers of differential migration as predicted by the body size, social dominance or arrival time hypothesis^30–32^. The GPS tracking data presented in this study revealed that lager birds tend to arrive earlier at the breeding sites. Larger birds, in particular males, may benefit from a timely arrival at the breeding sites by securing better breeding sites^32^. However, the time of arrival did not differ between sexes, nor did this cause differences in wintering area according to body size (Table 1). Instead, sex-dependent differences in migration only related to the timing of autumn migration and time spent at stopover sites (Fig. 3).

During the past decade, high-resolution tracking has revealed individual differences in the timing, routes and stopover sites between autumn and spring migration in several bird species^36,37^. These differences have been linked to factors such as a higher selective pressure for a timely arrival at the breeding sites in spring, or different energetic constraints between autumn and spring^18,22,34^. Such energetic constraints may relate to the annual moult. Breeding, moulting and migration generally succeed or overlap with each other within the annual cycle of most bird species, although not necessarily in this order^18^. This might create a peak in energy demand during late summer and autumn, especially when moult and migration overlap, which imposes energetic constraints that may lead to shorter migration bouts and/or an increased requirement for refuelling stopovers in autumn^7,22,38–40^.

The observed shifts in habitat use, and the occurrence of sex differences in the time course of autumn migration, indeed coincided with the timing of moult in our study population. Moult in Lesser Black-backed Gulls of the subspecies *intermedius* and *graellsii* commonly starts during, or shortly after, breeding and continues uninterruptedly until completion at the wintering area^41^. Autumn migration in these subspecies is characterised by a series of short hops and detours, spending a considerable amount of time on stopover sites before reaching their wintering sites along the western coasts of Europe and Africa^30,31^. This protracted migration and frequent use of stopover sites may, therefore, be driven by the temporal spike in energy requirement due to overlap between moult and migration or breeding. This interpretation is further supported by the fact that both subspecies differ markedly from the nominate *fuscus* in their timing of moult and migration^29^. In the latter subspecies, moult often only commences at its wintering grounds, and hence more rarely overlaps with migration. Moreover, individuals that have already started moulting at the breeding grounds suspend it during migration^42^. As predicted from an energy-budget perspective, such temporal separation of moult and migration may enable individuals of *fuscus* to make long migration bouts with only few, short stopovers on the way to their wintering areas along the East-African coast^43^.

Intriguingly, the females of our study population had already started to specialise on agricultural habitats from June onwards, one month prior to the onset of migration, and remained strongly tied to those habitats until the end of autumn migration (Table 3). Earlier GPS tracking studies in the Netherlands revealed comparable sex differences, whereby males on average specialised more on marine habitats throughout the breeding season, whereas females specialised more on agricultural habitats, in particular during chick rearing^44^. Our results, in addition, revealed that these sex differences in foraging strategies are not alleviated by the time chicks fledge and adults commence their autumn migration. Rather, differences in habitat use between males and females persisted during autumn migration until both sexes reached their wintering grounds, and as such coincide with the full extent of the complete post-breeding moult^29^. Such differences in habitat use may originate from intra-specific resource competition or balanced food provisioning within pairs during chick rearing, with females being more efficient in exploiting agricultural habitats^44^. Females, therefore, probably benefit more from exploiting agricultural habitats along autumn migration, also because such habitat is less available at the wintering grounds.

Alternatively, or in addition, foraging in marine environments involves a substantial amount of flight behaviour to track and compete for fishery discards. Such discards are, therefore, more energy demanding to obtain than terrestrial food sources, but also have a higher energy content^45–47^. Given their larger body size, males may be more successful as marine foragers, either because of their higher competitiveness, or because of their longer wings, as foraging at sea involves increased foraging ranges^44,48^. Wing length/load then again may not only play a role for marine foraging, but will also affect flight efficiency under different wind conditions, which might differ between habitats as well as between sexes^49,50^. Because of the higher energy content of fish discards, predominant marine foragers may build up more fat prior to migration and/or refuel more efficiently on migration stopovers, which may reduce the need for stopovers during migration^51,52^. However, as sexes invest equally in raising their offspring, and because adults depart from their breeding colony shortly after their young have fledged, it appears unlikely that differences in condition at departure would impact the timing and habitat use during autumn migration.

Individual specialisation may not only relate to competitive differences, but may also be driven by differences in nutritional requirements^53^. For example, females may have different nutritional requirements after egg laying or may prefer more reliable, yet less profitable, agricultural fields over a high-risk high-gain marine foraging strategy. Moreover, foraging specialisation may be affected by moult, as a reduced flight performance^7,40^ could temporarily alter competitive ability.

Understanding the degree to which female specialisation on terrestrial feeding results from competition avoidance or reflects higher female preferences to exploit such resources, and how selection pressures across the life-cycle shape such sexual segregation, requires individual-based analyses on daily foraging decisions and remains a topic for further research. Unfortunately, no observational data are currently available to assess whether, and how, competition avoidance acts as driving force behind the observed differential migration.

Taken together, our study demonstrates that high-resolution tracking is essential to understand how variation in migration strategies can arise from trade-offs throughout the annual cycle. In our study population of Lesser Black-backed Gulls, we found consistent sexual differences in both the time course and habitat use during autumn migration. Such differences were no longer evident in the wintering grounds, as could be expected if these are primarily driven by energetic constraints due to overlap between moult and breeding or migration. Similarly, no sex differences occurred during spring migration, which was only interrupted by a limited number of short stopovers. Based on these results, we propose that overlap between three major energy-demanding activities creates a temporal window in autumn when females seem to benefit from specialising on agricultural food sources and prolonging their autumn migration. Sex differences in migration thereby occur at small spatial scale at stopover sites, through differences in timing and habitat use during autumn migration.

## Materials and Methods

### GPS tracking

Between June 2013 and June 2017, 107 adult (i.e. at least four years of age) Lesser Black-backed Gulls were fitted with 18 g UvA-BiTS GPS trackers (see Bouten *et al*.^54^ for technical details). Trackers were attached using a harness of Teflon ribbon threaded with a nylon string. All individuals were caught on their nests during incubation at the colonies of Ostend (51.23N 2.93E, 3 males and 3 females), Zeebrugge (31.35N 3.17E, 33 males and 37 females) or Vlissingen (51.45N 3.69E, 18 males and 13 females) using walk-in traps or clap nets. Birds were weighed, measured (wing, tarsus, head, bill and gonys length), sexed, and fitted with metal and colour rings. All individuals were sexed molecularly^55^.

The UvA-BiTS tracking system is solar powered and allows bi-directional communication with a ground base station, remotely downloading data and uploading new settings^54^. GPS trackers are equipped with different sensors, enabling the collection of a variety of measurements including altitude and tri-axial acceleration. In this study we only use positional and ground speed data provided by the GPS. During the breeding season, GPS fixes were taken every 3 minutes on average (although settings could range within 1 and 30 minutes) with at least 60 m accuracy^54^. However, as base stations were positioned at the breeding colonies, the GPS resolution was set between 10 and 30 minutes outside the breeding season to prevent memory overflow. A full description of the obtained data and quality control is provided in Stienen *et al*.^56^. Birds were monitored throughout the breeding season, and breeding success (eggs, hatched chicks or fledged chicks) was recorded where possible. The deployment of GPS trackers was authorized by the ethical committee for animal experiments of the University of Antwerp (ID numbers CDE2013–73 for Ostend and Zeebrugge, and ECD2015–67 for Vlissingen) and conducted in accordance to Flemish, Belgian and Dutch legislation.

### Data processing

The GPS positions of each individual were split into trajectories, which were subsequently classified in relation to the different events within a bird’s annual cycle: breeding, autumn migration, autumn stopover, wintering, spring migration and spring stopover (an elaborated example is provided in Fig. S1). Trajectories consisted of all GPS positions between a departure from, and the next arrival at, a resting site. A resting site is thereby defined at a 0.01° resolution as a location where an individual spends at least 4 consecutive hours. During this interval, the individual should be at rest at least 90% of the time, meaning that it has a ground speed of maximal 1 m s^−1^ (to still allow for small movements such as drifting on a lake or at sea; Shamoun-Baranes *et al*. 2011). Trajectories thus start when a gull leaves a resting site and stops when it subsequently arrives again at a resting site with a ground speed of maximal 1 m s^−1^ (to prevent termination of a trajectory by possible fly-bys).

To classify trajectories, first we determined for each year when a bird first and last visited the breeding area. The breeding area was defined as a polygon enclosing the three breeding colonies where the gulls were captured (Ostend, Zeebrugge and Vlissingen). All trajectories for an individual between these dates were classified as summer trajectories. Note that these trajectories comprised both foraging trajectories during active breeding and wandering behaviour, which adult Lesser Black-backed gulls are known to undertake before incubation, after fledging of the chicks, after a failed breeding attempt^30^ or during a year of non-breeding (pers. obs.). The first and last visit to the breeding area thus corresponds to the end of spring migration and onset of autumn migration, respectively. Note that since all trackers were placed during the breeding season, only the start of autumn migration could be determined during the first year of tracking. Next, for each year and each bird the wintering area was determined. Lesser Black-backed Gulls are known to winter in a restricted area in which they use several resting sites on a regular basis^6,31^. We therefore first clustered resting sites. All sites visited between returning visits to any other site within two months (i.e. 62 days) were assumed to belong to the same cluster. This generally yielded multiple clusters, as birds tend to have long stopovers during migration where they return to roost for several consecutive nights. Hence, the wintering area was defined as the furthest cluster from the breeding area where birds spent at least 14 consecutive days (Fig. S1). This criterion yielded a single, unambiguous wintering area for each year and individual. All trajectories between the first (i.e. end of autumn migration) and last (i.e. start of spring migration) visit to a resting site in the wintering area were consequently considered as wintering trajectories. Spring or autumn migration and stopover trajectories thus encompassed all trajectories between the last visit to the wintering area and first visit to the breeding area, or vice versa. Trajectories were considered as migration when they started for the last time at a particular resting site (i.e. directional movement), whereas stopover trajectories were those that started from a resting site which was revisited before the bird arrived in its wintering or breeding area.

Habitat use was calculated for each GPS position within a trajectory where a bird was not in active flight. Recorded ground speeds of Lesser Black-backed Gulls showed a bimodal distribution with modes separated by a minimal density at about 4.5 m s^−1^ (16.2 km h^−1^, Fig. S2). Speeds above 4.5 m s^−1^ were therefore assumed to correspond to active flight^48^. Speeds below 4.5 m s^−1^ encompassed a variety of behaviours, including standing, resting, walking, floating, soaring and tortuous flight (e.g. when foraging behind a fishing vessel). Hence birds were assumed to actively use a habitat when ground speed fell below 4.5 m s^−1^. For these GPS locations on a trajectory, the habitat type was assigned according to the Envisat 2015 ESA land cover database (300 m resolution)^57^. For analytic purposes, we regrouped the ESA land cover data into four categories: agricultural, urban, freshwater and marine habitats. Agricultural habitats comprised ‘rain fed cropland’, ‘irrigated cropland’, ‘mosaic cropland’ and ‘grassland’. Urban habitats comprised ‘urban’ and ‘bare area’. Freshwater habitats comprised ‘flooded tree cover fresh- or brackish water’, ‘flooded shrub or herbaceous cover’ and ‘water bodies’ that lie at least 2 km inland, while marine habitats comprised ‘flooded three cover saline water’ and all other ‘water bodies’. Other land cover types such as forests were not assigned to any of these four categories and were omitted from the analysis as they jointly represented less than 0.5% of the observations, and are not known to be used by our study species.

### Data analysis

We limited our analysis to complete migration routes (i.e. successful arrival at the breeding or wintering area) for which we had at least 1 GPS location per hour. Using this criterion, a total of 150 migration routes of 48 individuals (22 males, 26 females) were retained between autumn 2013 and spring 2017: i.e. 31 autumn migration routes of 19 males, 39 autumn migration routes of 21 females, 35 spring migration routes of 22 males and 45 spring migration routes of 24 females.

Linear mixed effects models were fitted to the data to test for differences in migration distance and timing of migration (i.e. start and end date) in relation to sex and body size. The day at which spring and autumn migration started or ended was expressed as a numeric value, the first of January being day 1. Migration distances were calculated as the Haversine distance between breeding and wintering area. Body size was expressed as a body size index, which explained 80% of the variation in body size in our study population (Fig. S3)^32^:

1 $$0.86\times tarsus\,length+0.95\times head\,length+0.90\times bill\,length+0.88\times gonys\,width$$

All measurements are expressed in mm. All models included body size and sex and the two-way interactions as fixed effects. Because migration distance may be confounded with timing and/or duration of migration, migration distance and the two-way interactions with body size and sex were added as additional fixed effects when testing for differences in timing of migration. Individual and tracking year were added as random effects to all full models. The level of significance of both random effects was assessed using likelihood-ratio tests, and only tracking year was retained in the final model.

Similarity in habitat use between pairs of individuals was calculated based on the Bray-Curtis similarity index^58^:

2 $$Similarit{y}_{m,1-2}=1-\frac{{\sum }_{i}|{p}_{m,i,individual1}-{p}_{m,i,individual2}|}{2}$$

*P*_*m*,*i*_ represents the fraction of GPS observations of an individual assigned to habitat type *i* (agricultural, urban, freshwater or marine) during a given month (*m*). A value of 1 thus corresponds to identical habitat use, whereas a value of zero corresponds to complete segregation. To test if intra- and inter-sex variation in habitat use changed throughout the year, a linear mixed effects model was fitted including month and type of comparison (i.e. male-female, male-male or female-female) and the two-way interaction as fixed effects. To account for statistical dependence, the combination of individuals being compared was included as a random effect and a continuous first order autocorrelation structure was fitted. Similarities in habitat use were pooled per month across years. However, to fit a continuous autocorrelation structure, each month between June 2013 and May 2017 was assigned a unique, consecutive numeric value (June 2013 being month 1). The level of significance of random effects and autocorrelation structures was assessed using likelihood-ratio tests. The fraction of time spent in agricultural, urban, fresh water and marine habitats was compared between males and females by fitting a linear mixed effects model that included month and sex as fixed effects, individual as a random effect and a first order continuous autocorrelation structure as described above.

Only significant two-way interactions were retained in models, whereas main effects were always retained to avoid parameter estimation bias^59^. Finally, normality, independence and homoscedasticity of model residuals was inspected (Figs S4–S12)^60^. All analyses were carried out in R using the nlme package^61,62^. Great circle distances were calculated using the distHaversine function in the geosphere package^63^. Maps were created using the packages maps, mapData and OpenStreetMap^64–66^.

### Data availability

 A subset of the data is available at http://doi.org/10.15468/02omly.

## Electronic supplementary material

Supplementary information
