# Supplementary material for: High-resolution GPS tracking reveals sex differences in migratory behaviour and stopover habitat use in the Lesser Black-backed Gull Larus fuscus
Source: Sci Rep. 2018 Mar 29;8:5391. doi: 10.1038/s41598-018-23605-x (PMC5876360; doi:10.1038/s41598-018-23605-x)
Supplement: Supplementary file 1 — Supplementary information [file 41598_2018_23605_MOESM1_ESM.docx]

Supplementary information for:

High-resolution GPS tracking reveals sex differences in migratory behaviour and stopover habitat use in the Lesser Black-backed Gull *Larus fuscus*

Jan M. Baert^1,2^, Eric W. M. Stienen^3^, Brigitte Heylen^1,2^, Marwa M. Kavelaars^1,2^, Roland-Jan Buijs^4^, Judy Shamoun-Baranes^5^, Luc Lens^2^ and Wendt Müller^1^

^1^ Behavioral Ecology and Ecophysiology Research group, University of Antwerp, Universiteitsplein1, 2610 Antwerp, Belgium

^2^ Terrestrial Ecology Unit (TEREC), Ghent University, K.L. Ledeganckstraat 35, 9000 Ghent, Belgium

^3^ Research Institute for Nature and Forest (INBO), Havenlaan 88 box 73, 1000 Brussels, Belgium

^4^ Buijs Eco Consult B.V., Philips van Dorpstraat 49, 4698 RV Oud-Vossemeer, The Netherlands

^5^ Institute for Biodiversity and Ecosystem Dynamics (IBED), University of Amsterdam, P.O. Box 94248, 1090GE Amsterdam, The Netherlands

**
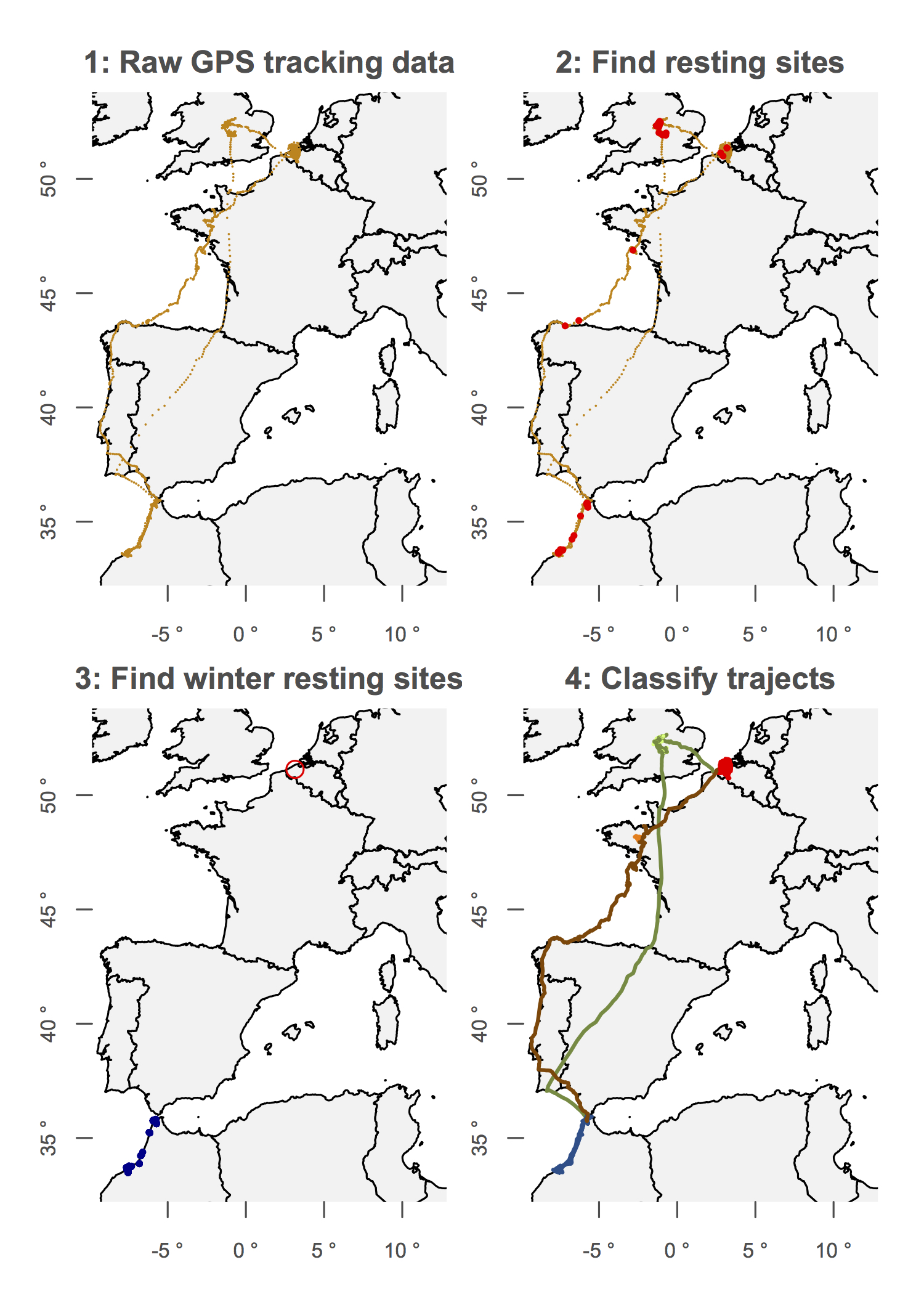
**

**Figure S1:** Illustration of the classification of GPS data into trajectories for one year of data of the birds (‘Ella’). First, from the raw data (panel 1) resting sites (red dots) are identified (panel 2). Next resting cells are clustered in to groups of resting sites that are recurrently visited by the bird. The wintering area (panel 3) is than defined as the cluster of resting cells (blue dots) furthest from the breeding area (red circle). Based on the identification of the winter area, and stopover resting cells, trajectories are classified (panel 4). Summer trajectories are indicated in red, autumn migration trajectories are indicated in dark green, autumn stopover trajectories are indicated in light green, winter trajectories are indicated in blue, spring migration trajectories are indicated in brown and spring stopover trajectories are indicated in orange. Maps are generated using the Maps package in R^1,2^.

**Figure S2:** Upper panel: Histogram of the ground speed distributions of all tracked Lesser Black-backed Gulls. The dotted red line indicates the 4.5 m s^-1^ cut-off speed under which birds were assumed to use the surrounding habitat. Lower panel: illustration of the determination of habitat used of one of the birds ('Joke') during winter. Lines represent trajectories and red dots GPS fixes where the ground speed was lower than 4.5 m s^-1^. The map is generated using the OpenStreetMap package in R^2,3^ under the  Open Data Commons Open Database License (www.openstreetmap.org/copyright).

**Figure S3:** Boxplots of the body size index values for both sexes.

**Figure S4:** Model residuals for the linear mixed effects model predicting the migration distance.

**Figure S5:** Model residuals for the linear mixed effects model predicting the onset of autumn migration.

**Figure S6:** Model residuals for the optimal linear mixed effects model predicting the end of autumn migration.

**Figure S7:** Model residuals for the optimal linear mixed effects model predicting the onset of spring migration.

**Figure S8:** Model residuals for the optimal linear mixed effects model predicting the end of spring migration.

**
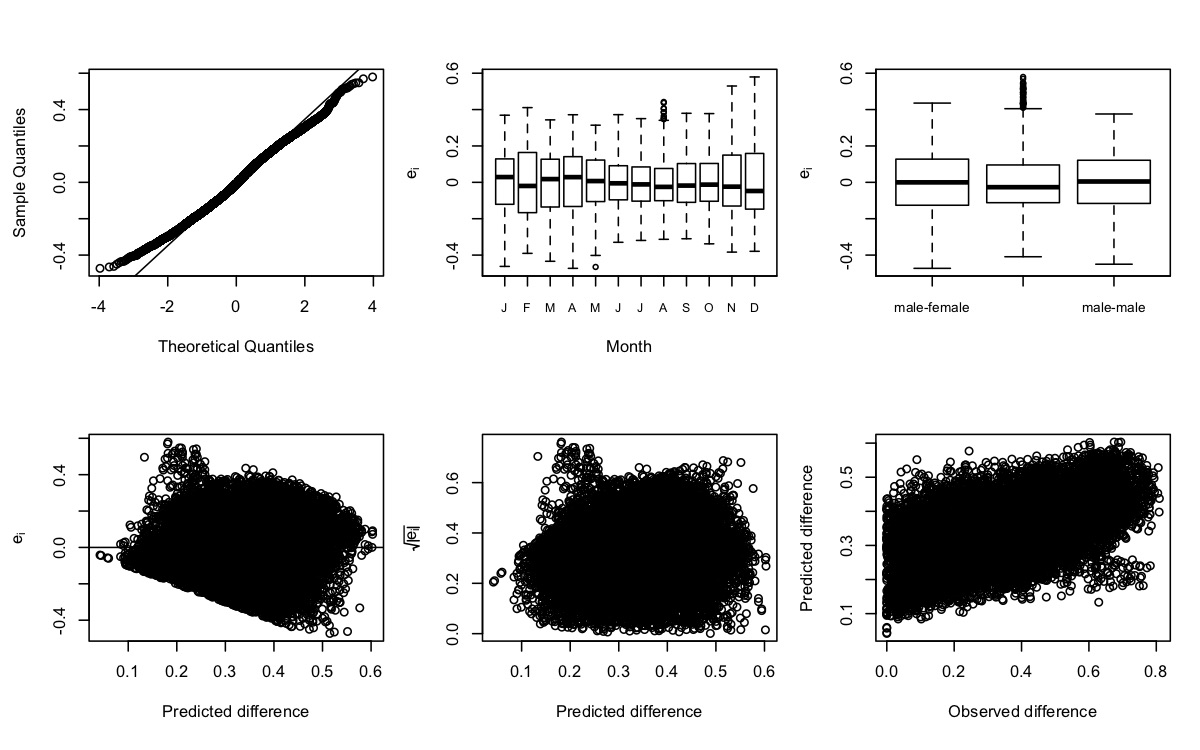
**

**Figure S9:** Model residuals for the linear mixed effects model predicting differences in habitat use.


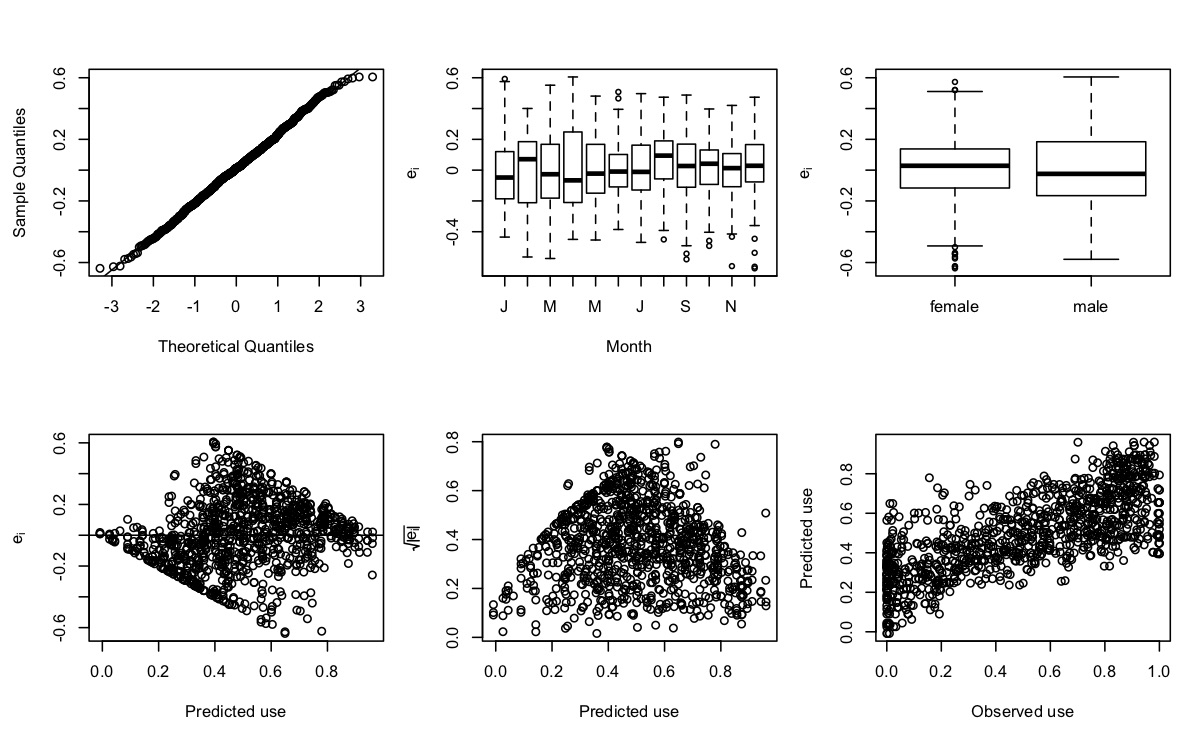


**Figure S10:** Model residuals for the optimal linear mixed effects model predicting the use of agricultural habitat.

**
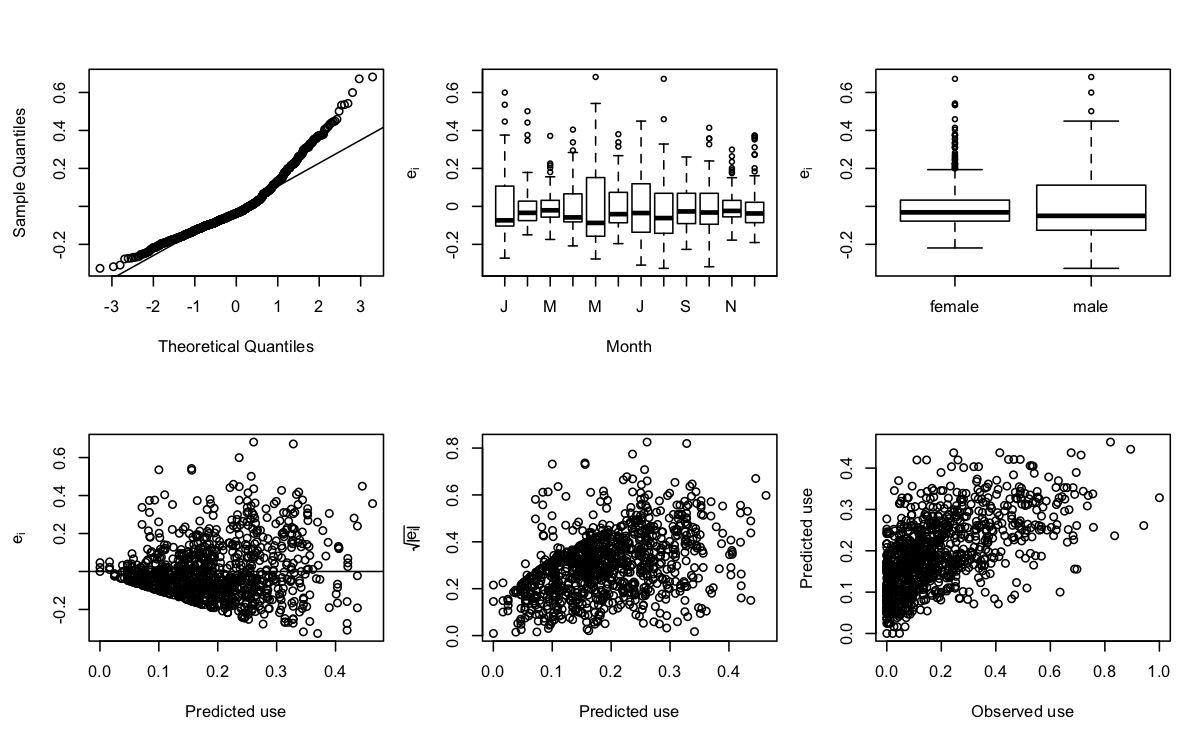
**

**Figure S11:** Model residuals for the optimal linear mixed effects model predicting the use of urban habitat.


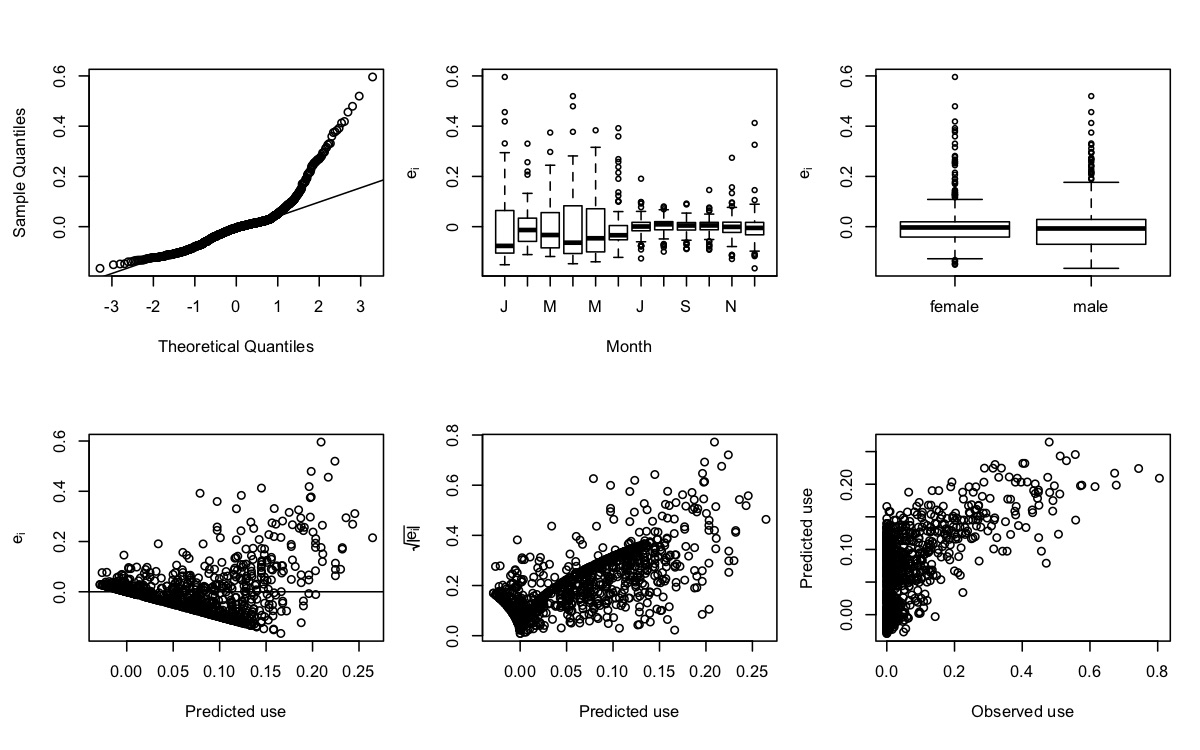


**Figure S12:** Model residuals for the optimal linear mixed effects model predicting the use of freshwater habitat.

**
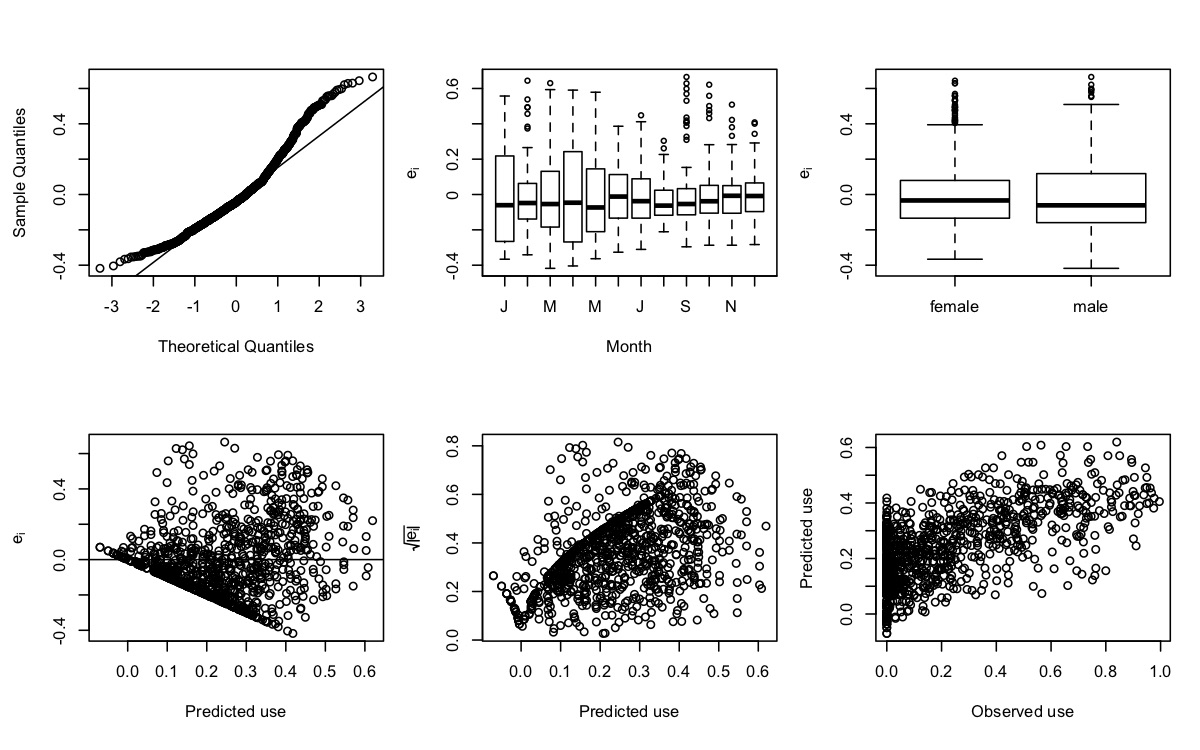
**

**Figure S13:** Model residuals for the optimal linear mixed effects model predicting the use of marine habitat.

**Figure S14:** Boxplots indicating the start and end dates for autumn and spring migrations for birds of which the nest was no observed (NO), only laid eggs (EGG), of pulli hatched (PUL) or successfully fledged (FLE).

**References**

1. Becker, R. A. & Wilks, A. R. maps: Draw Geographical Maps. (2016).

2. R development core Team. R: A language and environment for statistical computing. R Foundation for Statistical Computing. (2017). Available at: http://www.r-project.org/.

3. Stotz, J. P. OpenStreetMap: Access to open street map raster images. (2016). Available at: https://CRAN.R-project.org/package=Openstreetmap
